# Editorial: Robotic and video-assisted surgery for cancer treatment

**DOI:** 10.3389/fonc.2024.1448143

**Published:** 2024-08-29

**Authors:** Gianluca Rompianesi

**Affiliations:** Hepato-Bilio-Pancreatic, Minimally Invasive, Robotic and Transplant Surgery Unit, Federico II University Hospital, Naples, Italy

**Keywords:** robotic surgery, laparoscopic surgery, thoracoscopic surgery, minimally invasive surgery, cancer surgery

In the last two decades, the rapid advancement and dissemination of robotic- and video-assisted surgeries have revolutionised the surgical landscape. These minimally invasive approaches offer several advantages over traditional open surgery such as smaller incisions, high control thanks to a magnified three-dimensional vision, and exceptional precision due to the filtration of the physiological tremor and a greater range of motion than a human hand (1–3). Moreover, the robotic platform can help surgeons enhance their performances and reduce physical strain and fatigue by providing ergonomic advantages. All of these can translate into better patient outcomes in terms of less trauma to the body and pain, reduced blood loss, lower risk of infection, faster recovery times, and shorter length of hospital stay (4).

These developments have contributed to the advent of a new precision medicine era thanks to the possibility of integrating artificial intelligence (AI) and automation and performing telesurgery.

While robotic- and video-assisted surgeries have been proven to be effective, they also have several limitations. These technologies require specialised training and can be expensive to implement, which can limit access for some patients. Additionally, there are certain cancers and surgical procedures that may not be suitable for these techniques.

Robotic- and video-assisted surgeries have been progressively implemented in cancer patients’ treatment and are currently playing a major role in almost all surgical oncology fields.

This Research Topic focuses on novel applications of robotic- and video-assisted surgeries for cancer treatment with a comprehensive overview of the most up-to-date techniques.

Urology has been one of the surgical oncology specialties that pioneered the adoption of robotic surgery, with the first robotic-assisted prostatectomy performed in 2000 by Binder and Kramer. Localised prostate cancer can be effectively treated with robotic radical prostatectomy, but a possible complication that can negatively affect the quality of life of patients is postoperative urinary incontinence. Li et al. in their report investigated the role of anatomic reconstructed technique for periurethral structures during robotic-assisted radical prostatectomy. This novel technique proved to be effective in improving 1- and 3-month continence compared to the control group.

A possible fascinating breakthrough in robotic surgery is the development of AI-integrated systems capable of performing autonomous and semi-autonomous tasks, further enhancing the safety and accuracy of surgical performances. Several urological diseases and conditions can be currently treated with a minimally invasive robotic approach, including bladder cancer (BCa). This rapidly evolving scenario originates a fervent scientific activity in the field, as shown by Zhou et al. in their bibliometric analysis on the application of artificial intelligence in BCa, where most of the references with the citation bursts were related to robotic-assisted surgery.

In the area of oncologic digestive surgery, rectal cancer represents a frequent and challenging entity, affecting approximately 40% of patients with colorectal tumours and with complex treatment options and difficult surgical anatomy. In such a scenario, the accuracy of the robotic platform has the potential to provide significant advantages compared to the laparoscopic approach. Zhang et al. conducted a meta-analysis on 11,686 patients to evaluate the differences in postoperative short-term outcomes between robotic and laparoscopic resection of rectal cancer after neoadjuvant therapy. Despite increasing the operative time, the robotic approach emerged superior in reducing the risk of the conversion to open surgery and improving the total mesorectal excision (TME) incomplete rate.

The TME during rectal cancer surgery allows for lower rates of local recurrence and increased anal sphincter and nerve sparing. Guo et al. retrospectively analysed a cohort of 533 patients undergoing minimally invasive TME. The authors constructed and validated a four-variable nomogram predicting the TME difficulty and demonstrated that the robotic-assisted technique was superior in terms of lower rate of diverting ileostomy, lower estimated blood loss, and shorter postoperative hospital stay in the most challenging cases.

Oesophageal cancer is a fearsome entity burdened by high malignancy and a poor prognosis. The surgical excision of the primary tumour and the regional lymph nodes represents the only curative option. Minimally invasive esophagectomy has been shown to reduce surgical damage to the chest and abdominal wall and to lower postoperative complications while providing long-term survival results equivalent to those of open surgery. Xue et al. retrospectively compared two groups of patients receiving video-assisted minimally invasive or robotic esophagectomy. The robotic approach provided better results in terms of more complete lymphadenectomy, shorter operating time, reduced bleeding, and reduced postoperative discomfort.

Video-assisted thoracoscopic surgery is being widely adopted to treat benign oesophageal tumours, such as leiomyomas. This technique was adopted by Ding et al. to successfully perform the simultaneous resection of an oesophageal leiomyoma and a pulmonary segmentectomy for mixed ground-glass opacity.

A further application of the robotic technique is the transoral robotic surgery, adopted in cases of oropharyngeal cancer. Ji et al. reported satisfactory results in their retrospective analysis of 41 patients with oropharyngeal squamous cell carcinoma and undergoing transoral robotic surgery, with no observed perioperative mortality or severe complications and favourable long-term functional outcomes.

The most frequent gynaecological malignancy is cervical cancer. When diagnosed in the early stages, usually radical hysterectomy is the preferred treatment option usually in fit patients. Dai et al. performed a meta-analysis of prospective cohort studies or randomised controlled trials comparing robotic and laparoscopic radical hysterectomies for treating cervical cancer. Robotic surgery appeared to offer several advantages compared to laparoscopy, including reduced blood loss, a greater number of dissected pelvic lymph nodes, and a shorter hospital stay.

Inadvertently retained cotton foreign bodies after surgery are usually referred to as gossypibomas, and if not promptly excised, they can create a mass-forming reaction that can sometimes be mistaken for a tumour as reported in approximately 20 cases in the literature. Han et al. reported a case of a successful laparoscopic video-assisted excision of a gossypiboma initially diagnosed as a jejunal tumour.
